# Identification of miR-20a-5p as Robust Normalizer for Urine microRNA Studies in Renal Cell Carcinoma and a Profile of Dysregulated microRNAs

**DOI:** 10.3390/ijms22157913

**Published:** 2021-07-24

**Authors:** Julia Oto, Raquel Herranz, Emma Plana, José Vicente Sánchez-González, Javier Pérez-Ardavín, David Hervás, Álvaro Fernández-Pardo, Fernando Cana, César David Vera-Donoso, Manuel Martínez-Sarmiento, Pilar Medina

**Affiliations:** 1 Haemostasis, Thrombosis, Arteriosclerosis and Vascular Biology Research Group, Medical Research Institute Hospital La Fe (IIS La Fe), 46026 Valencia, Spain; juliaotomartinez@gmail.com (J.O.); raq.herranz@gmail.com (R.H.); plana_emm@gva.es (E.P.); alvarofernandezpardo@gmail.com (Á.F.-P.); fernando_cana@iislafe.es (F.C.); 2 Angiology and Vascular Surgery Service, La Fe University and Polytechnic Hospital, 46026 Valencia, Spain; 3 Department of Urology, La Fe University and Polytechnic Hospital, 46026 Valencia, Spain; josevicente.sg@gmail.com (J.V.S.-G.); ardavin.j@gmail.com (J.P.-A.); cdveradonoso@gmail.com (C.D.V.-D.); mmarsar@gmail.com (M.M.-S.); 4 Data Science, Biostatistics and Bioinformatics Unit, Medical Research Institute Hospital La Fe (IIS La Fe), 46026 Valencia, Spain; ddhervas@yahoo.es; 5 Department of Applied Statistics, Operations Research, and Quality, Universitat Politècnica de València, 46022 Valencia, Spain

**Keywords:** renal cell carcinoma, biomarker, normalizer, urine, miRNA, diagnosis, liquid biopsy

## Abstract

Renal cell carcinoma (RCC) is the third most frequent urinary malignancy and one of the most lethal. Current diagnostic and follow-up techniques are harmful and unspecific in low-grade tumors. Novel minimally invasive markers such as urine microRNAs (miRNAs) are under study. However, discrepancies arise among studies in part due to lack of consent regarding normalization. We aimed to identify the best miRNA normalizer for RCC studies performed in urine samples together with a miRNA profile with diagnostic value and another for follow-up. We evaluated the performance of 120 candidate miRNAs in the urine of 16 RCC patients and 16 healthy controls by RT-qPCR followed by a stability analysis with RefFinder. In this screening stage, miR-20a-5p arose as the most stably expressed miRNA in RCC and controls, with a good expression level. Its stability was validated in an independent cohort of 51 RCC patients and 32 controls. Using miR-20a-5p as normalizer, we adjusted and validated a diagnostic model for RCC with three miRNAs (miR-200a-3p, miR-34a-5p and miR-365a-3p) (AUC = 0.65; Confidence Interval 95% [0.51, 0.79], *p* = 0.043). let-7d-5p and miR-205-5p were also upregulated in patients compared to controls. Comparing RCC samples before surgery and fourteen weeks after, we identified let-7d-5p, miR-152-3p, miR-30c-5p, miR-362-3p and miR-30e-3p as potential follow-up profile for RCC. We identified validated targets of most miRNAs in the *renal cell carcinoma* pathway. This is the first study that identifies a robust normalizer for urine RCC miRNA studies, miR-20a-5p, which may allow the comparison of future studies among laboratories. Once confirmed in a larger independent cohort, the miRNAs profiles identified may improve the non-invasive diagnosis and follow-up of RCC.

## 1. Introduction

Renal cell carcinoma (RCC) represents 2–3% of all cancers and accounts for approximately 90% of all kidney malignancies. Moreover, it is one of the most lethal urological malignancies. RCC is twice more frequent in men than in women and is mainly diagnosed between the fourth and six decade of life. The initial symptoms of RCC may be unspecific and belated [1], thus increasing mortality and causing approximately 30% of patients to debut with a paraneoplastic syndrome. In fact, seldom is the classic triad of flank pain, palpable abdominal mass and frank hematuria observed (6–10%). Conversely, the widespread use of abdominal imaging techniques for other medical purposes has increased the incidental detection of RCC, presently representing the primary form of new diagnoses. In addition, despite new treatment options, late-stage diagnoses have abysmally low survival rates [2].

The most prevalent subtypes of RCC are clear-cell RCC (ccRCC), the predominant RCC subtype accounting for 80% of all RCC; papillary RCC (papRCC, 15% RCC) and chromophobe (chrRCC, 5%) [3]. RCC management and prognosis differ among RCC subtypes [4]. At present, imaging techniques with contrast media such as contrast ultrasound, computerized tomography (CT scan) or magnetic resonance imaging (MRI) are employed to diagnose RCC, which is confirmed by the histopathological analysis of the specimen once removed. However, these techniques are invasive and sometimes harmful for the patient. Liquid biopsy is a revolutionary technique that allows the detection of circulating tumor cells, nucleic acids, and exosomes released by the tumor into the bloodstream or other biological fluids such as urine. Accordingly, efforts are being made to establish liquid biopsy as an alternative to tumor percutaneous biopsy, which is highly invasive, hazardous and cannot be performed when clinical conditions worsen or when the tumor is inaccessible [5,6]. In particular, urine represents the ideal sample to develop novel tools to diagnose RCC and monitor the follow-up and prognosis of RCC patients.

microRNAs (miRNAs) are small non-coding RNAs that regulate protein expression, and have been proposed as regulatory molecules and biomarkers in virtually all cancer types [7], including urological tumors [8,9,10]. miRNAs seem to have an important role in the maintenance of renal homeostasis and the development of kidney diseases. Moreover, miRNAs are dysregulated in the urine of patients with pathological kidney conditions [11,12] including RCC [13]. Accordingly, an RCC classification has been proposed based on the analysis of miRNAs in RCC tissue with high accuracy [14,15]. We recently reviewed previous studies that propose urine miRNAs as potential biomarkers for RCC [16] and we noticed that only a small number of miRNAs were shared by these studies, what is probably due to the lack of standardization in the protocols used among laboratories. Although differences may arise in the selection criteria of patients and controls, sample processing, RNA isolation protocols and miRNA quantification techniques employed, studies mainly differ in the normalization strategy used, what certainly represents a crucial step in any miRNA study and hampers the possibility of comparing the available literature. To date, no consensus exists on internal reference miRNAs for RCC studies performed in urine samples by Real Time quantitative Polymerase Chain Reaction (RT-qPCR); consequently, this became one of the main goals of the present study.

In our study we aimed to identify for the first time the best miRNA normalizer for miRNA studies in RCC conducted in urine samples. Secondly, employing the best miRNA normalizer, we aimed to identify a novel profile of miRNAs in urine able to distinguish RCC patients from controls. Thirdly, we aimed to find a profile of dysregulated miRNAs in the urine of RCC patients before and after surgery with potential follow-up value. The discovery of a good and reproducible internal miRNA normalizer will eliminate the current inconsistency among studies and will finally allow the comparison of urine miRNA studies in RCC. Then, the identification of non-invasive reliable biomarkers may improve RCC diagnosis, follow-up and prognosis, reducing the performance of harmful and expensive procedures.

## 2. Results

### 2.1. Clinical Characteristics of the Study Subjects

A total of 67 RCC patients were recruited and followed for one year. In the screening stage, samples from 16 ccRCC patients were analyzed. We decided to study exclusively the most predominant subtype of tumor (ccRCC) in order to analyze a homogenous group of patients. In the validation stage, we studied an independent cohort comprising 51 RCC patients (29 ccRCC, 16 papRCC and 6 chrRCC). Additionally, we studied 48 age- and sex-matched healthy volunteers (16 in the screening stage and 32 in the validation stage) and 13 benign renal angiomyolipomas in the validation stage. The clinical characteristics of the study subjects are depicted in Table 1.

### 2.2. Quality Internal Control with Synthetic Spike-in RNAs

**Figure 1:**
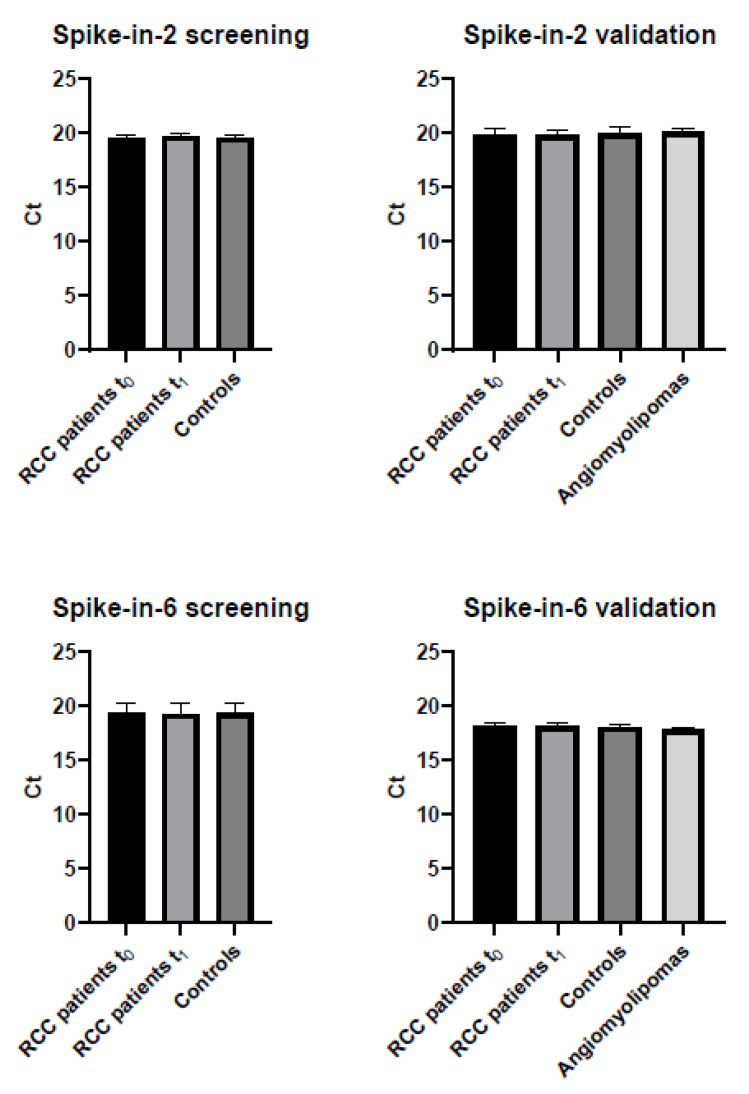
Differences in expression levels of the synthetic spike-in RNAs among the study groups. Spike-in 2 monitors the RNA isolation step and spike-in 6 monitors the retrotranscription efficiency. Expression levels are represented as Ct values and error bars represent standard deviation. ANOVA *p* > 0.05.

To ensure that miRNA quantification was not influenced by technical and interpersonal variability, synthetic non-human spike-in RNAs are frequently used. We assessed the RNA isolation step by adding the synthetic spike-in 2 RNA during all RNA isolations, and the retrotranscription efficiency by adding the spike-in 6 RNA in all retrotranscription reactions. No differences were observed in any spike-in studied among the study groups (Figure 1), thus indicating a proper performance of the isolation and retrotranscription steps.

### 2.3. Selection of Candidate miRNA Normalizers and Analysis of Their Stability

**Figure 2:**
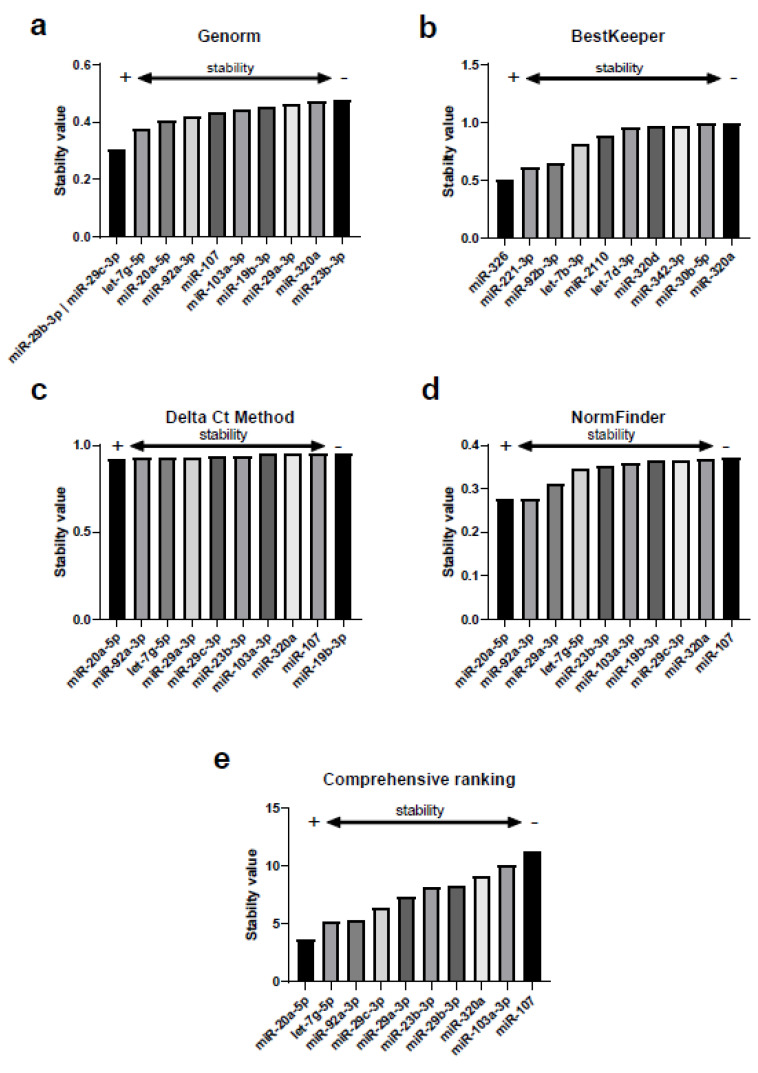
Selection of candidate miRNA normalizers and analysis of their stability conducted with the comprehensive tool RefFinder. Each graph represents the best 10 reference miRNAs selected by each algorithm: (**a**) Genorm, (**b**) BestKeeper, (**c**) Delta Ct method, (**d**) NormFinder and (**e**) Comprehensive raking. The lower the stability value, the higher the stability of each miRNA.

In the screening stage we analyzed a total of 179 miRNAs in each sample and obtained high quality signals in 120 of those miRNAs (mean of the Ct < 35) both in RCC patients and controls, thus these were included in the analysis with RefFinder. This tool integrates the computational algorithms Genorm, BestKeeper, Delta Ct and Normfinder. Figure 1 shows the best 10 reference miRNAs selected by each algorithm. The stability analysis conducted with Genorm revealed that the greatest stability was reached by the combination of miR-29b-3p and miR-29c-3p (Figure 2a). BestKeeper revealed that the most stable miRNA was miR-326 (Figure 2b). The Delta Ct method and Normfinder agreed with the most stable miRNA being miR-20a-5p (Figure 2c,d). Finally, the recommended comprehensive ranking that integrates all the previous algorithms, rendered miR-20a-5p as the most stable miRNA (Figure 2e), being aligned with the results of the Delta Ct method and Normfinder.

### 2.4. Differences in Expression Levels of the Candidate miRNA Normalizer between RCC Patients and Controls

**Figure 3:**
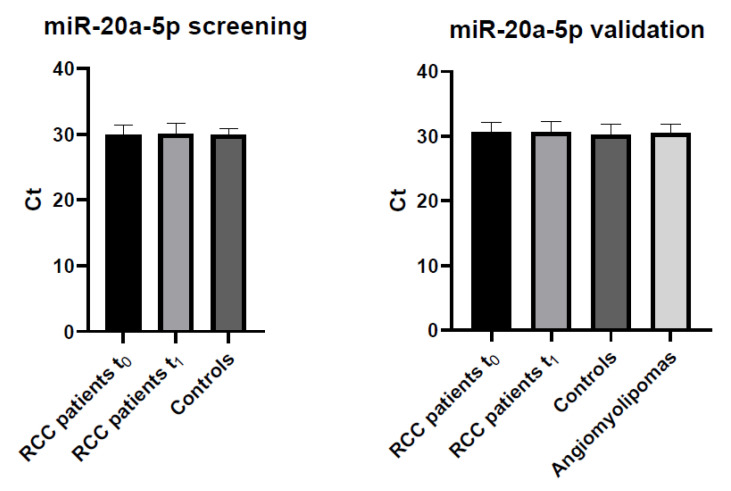
Differences in expression levels of the candidate miRNA normalizer miR-20a-5p selected by the comprehensive ranking of RefFinder between RCC patients and controls. Expression levels are represented as Ct values and error bars represent standard deviation. ANOVA *p* > 0.05.

A crucial characteristic of a good normalizer is the stable expression among the study groups analyzed. Accordingly, we compared the mean Ct values of the most stable miRNA proposed by the comprehensive ranking among the study subjects. As seen in Figure 3, no differences were observed in the expression of miR-20a-5p between RCC patients and controls studied in the screening or validation cohorts, or with angiomyolipomas (ANOVA *p* > 0.05). This observation, together with its good expression level (mean of the Ct values = 29.97), made miR-20a-5p as the best normalizer for our study.

### 2.5. Identification of Dysregulated miRNAs in RCC Patients before Surgery Compared to Controls

Once miR-20a-5p was verified as a robust normalizer for urine miRNA studies in RCC, we used it as endogenous control to identify a profile of dysregulated miRNAs in RCC patients before surgery (t_0_) compared to healthy controls.

In the screening stage, we adjusted a multivariable elastic net logistic regression model for the diagnosis of ccRCC with the miRNA expression levels in the urine of patients before surgery (t_0_) and controls. This model included three miRNAs: miR-200a-3p, miR-34a-5p and miR-365a-3p.

**Figure 4:**
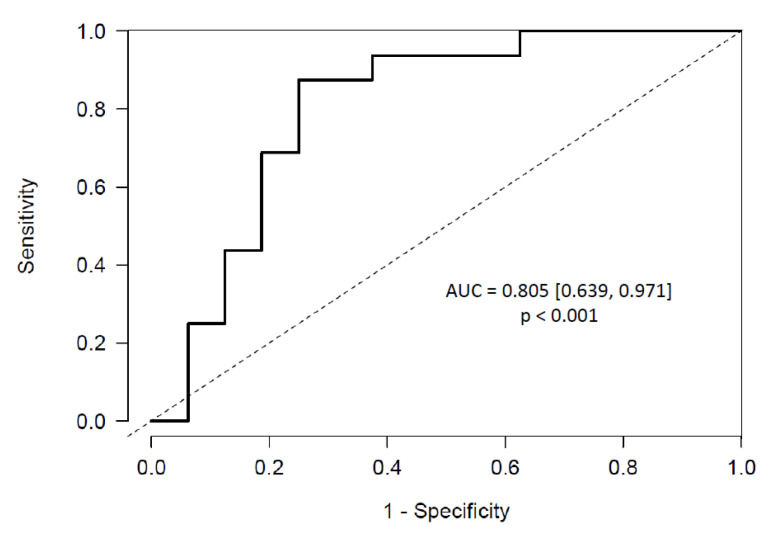
ROC curve of the multivariable elastic net predictive model for ccRCC that includes the expression of three miRNAs (miR-200a-3p, miR-34a-5p and miR-365a-3p) before surgery in urine.

The effect of each miRNA was stated as standardized odds ratios (ORs), reporting an increase of one standard deviation in the miRNA expression level. Accordingly, miR-200a-3p had standardized ORs of 1.01, miR-34a-5p of 1.15 and miR-365a-3p of 1.28, respectively. With this predictive model we achieved an area under the ROC curve (AUC) of 0.805 (Confidence Interval, CI 95% [0.639, 0.971], *p* < 0.001) (Figure 4).

The formula for estimating the risk of ccRCC with this model is as follows:

$$\mathrm{Pr}\left(Cancer\right)=\frac{e^{0.113-0.006*miR200a3p-0.135*miR34a5p-0.249*miR365a3p}}{1+e^{0.113-0.006*miR200a3p-0.135*miR34a5p-0.249*miR365a3p}}$$

**Figure 5:**
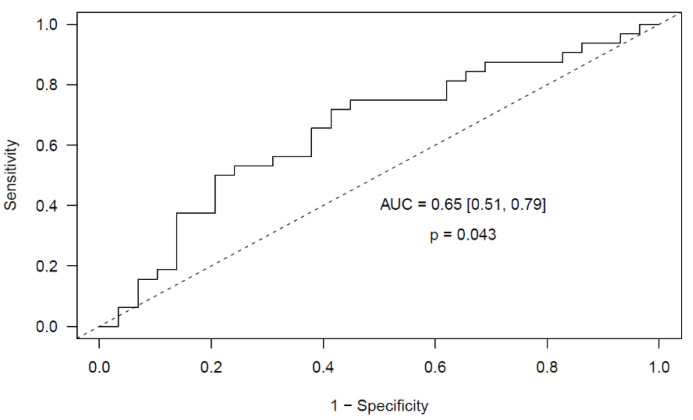
Validated ROC curve of the multivariable elastic net predictive model for ccRCC that includes the expression of three miRNAs (miR-200a-3p, miR-34a-5p and miR-365a-3p) before surgery in urine.

In the validation stage, we analyzed the expression of these three miRNAs in an independent cohort of ccRCC patients and we validated the predictive model achieving an AUC of 0.65 (95% CI [0.51, 0.79], *p* = 0.043) (Figure 5).

**Figure 6:**
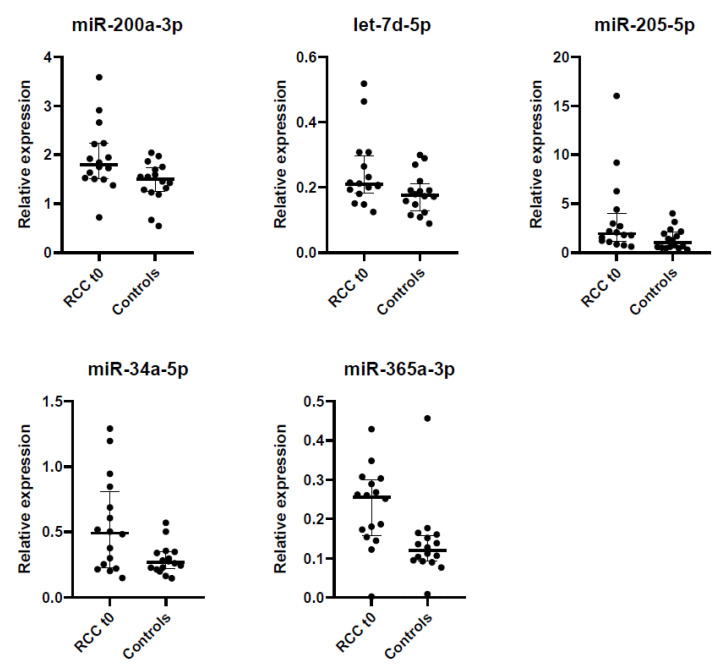
Relative expression of the five dysregulated miRNAs (miR-200a-3p, let-7d-5p, miR-205-5p, miR-34a-5p and miR-365a-3p) in ccRCC patients before surgery (t_0_) compared to controls.

Additionally, we identified those miRNAs dysregulated in patients before surgery compared to controls with the Wilcoxon-Mann–Whitney test. Fold-change (FC) expresses the ratio of the average expression level of a miRNA in ccRCC patients and controls. We identified five dysregulated miRNAs: miR-200a-3p (FC = 1.34, *p* = 0.024), let-7d-5p (FC = 1.38, *p* = 0.035), miR-205-5p (FC = 2.65, *p* = 0.029), miR-34a-5p (FC = 1.98, *p* = 0.038) and miR-365a-3p (FC = 1.68, *p* = 0.001) (Figure 6). Interestingly, miR-200a-3p, miR-34a-5p and miR-365a-3p were also predictors of ccRCC in the elastic net model.

Remarkably, there was a strong correlation among the miRNAs included in the predictive model and also among most dysregulated miRNAs between ccRCC patients and controls (Table 2).

No significant differences in the expression of these miRNAs were observed among ccRCC, papRCC and chrRCC (ANOVA *p* > 0.05), what indicates that the dysregulation of these miRNAs may not be specific of any RCC subtype. Additionally, a subgroup of 13 angiomyolipomas, a benign renal tumor, was analyzed. Interestingly, no differences were observed in the expression of these five miRNAs between healthy controls and angiomyolipomas (Wilcoxon-Mann–Whitney *p* > 0.05), suggesting that their dysregulation may be specific of RCC.

To evaluate whether the upregulation of these five miRNAs in the urine of RCC patients was influenced by tumor size, we conducted a regression model and no association was observed between tumor size and the expression of miR-200a-3p (R^2^ = 0.0115, *p* = 0.347), let-7d-5p (R^2^ = 0.0119, *p* = 0.339), miR-205-5p (R^2^ = 0.0493, *p* = 0.049), miR-34a-5p (R^2^ = 0.0016, *p* = 0.722) or miR-365a-3p (R^2^ = 0.0006, *p* = 0.832). Our results suggest that the presence of a RCC tumor might be sufficient to increase the expression of these miRNAs in urine, despite of the tumor grade.

### 2.6. Identification of Dysregulated miRNAs in RCC Patients before and after Surgery

**Figure 7:**
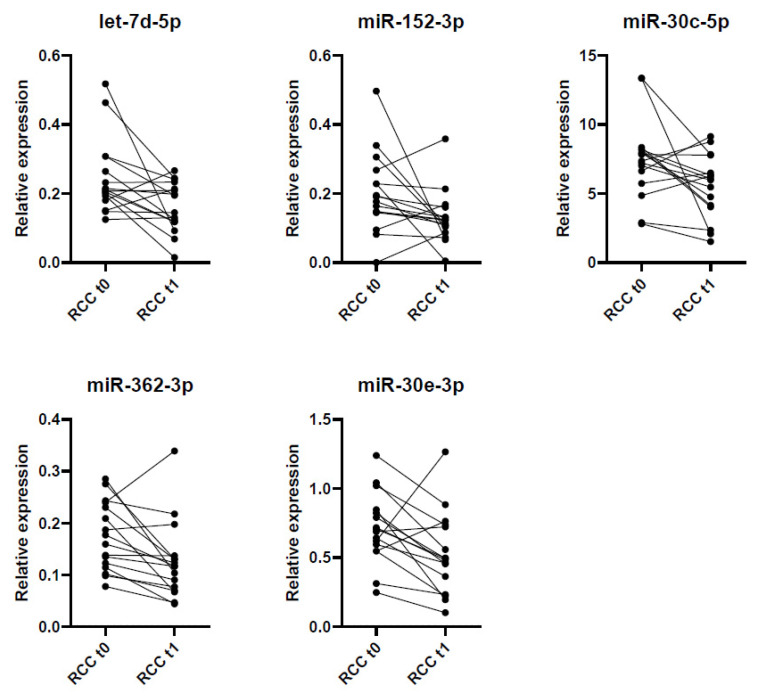
Relative expression of the five dysregulated miRNAs (**let-7d-5p**, **miR-152-3p**, **miR-30c-5p**, **miR-362-3p** and **miR-30e-3p**) in ccRCC patients before (t_0_) and after (t_1_) surgery.

In addition, we identified those miRNAs dysregulated before (t_0_) and fourteen weeks after surgery (t_1_) in the cohort of ccRCC patients of the screening stage with the paired Wilcoxon test. Fold-change expresses the ratio of the average expression level of a miRNA in t_0_ and t_1_. We identified five dysregulated miRNAs: let-7d-5p (FC = 1.53, *p* = 0.046), miR-152-3p (FC = 1.57, *p* = 0.023), miR-30c-5p (FC = 1.35, *p* = 0.042), miR-362-3p (FC = 1.37, *p* = 0.03) and miR-30e-3p (FC = 1.31, *p* = 0.048) (Figure 7). Importantly, a strong correlation was observed among most dysregulated miRNAs before and after surgery in ccRCC patients (Table 3).

### 2.7. Identification of miRNAs’ Targets

Once we selected the dysregulated miRNAs, we identified their validated and predicted target proteins related to RCC using the databases miRWalk 2.0 and Kyoto Encyclopedia of Genes and Genomes (KEGG) (Table 4). Noticeably, we identified an important number of validated targets for all miRNAs but miR-205-5p and let-7d-5p in the renal cell carcinoma pathway. Moreover, we further identified a group of predicted targets whose regulation by these miRNAs could be experimentally demonstrated in future studies.

## 3. Discussion

Novel non-invasive biomarkers are presently being investigated to circumvent several shortcomings in RCC diagnosis and monitoring such as the use of invasive or harmful techniques. Urine miRNAs, as part of liquid biopsy, have been explored as biomarkers for RCC diagnosis [16]. However, huge discrepancies arise among studies, to a great extent due to the nonexistence of normalization procedures [17]. In fact, to minimize the effect occasioned by methodology-related factors on miRNA expression levels, an accurate data analysis ought to be performed using appropriate normalizers for external and internal variation correction [18]. These normalizers should be chosen from a selection of candidates that are expected to be stably expressed over the entire range of samples being investigated, since miRNAs can be affected by the condition under study. Other normalization techniques, such as the use of the mean expression value of all commonly expressed miRNAs in a given sample, a combination of two miRNAs, other RNA species (snRNAs, snoRNAs and rRNAs) or exogenous synthetic RNAs, are strongly discouraged by the manufacturer. All in all, no consensus exists on a robust normalizer for urine studies in RCC.

In the present study, we aimed for the first time to ascertain the best miRNA normalizer for miRNA studies in the urine of RCC patients in order to avoid future inconsistencies among studies. We evaluated the performance of 120 candidate miRNAs with the comprehensive tool RefFinder, which integrates 4 programs (Genorm, BestKeeper, Delta Ct method and NormFinder) in 16 RCC patients and 16 healthy controls. We selected miR-20a-5p as the best normalizer for miRNA studies in urine of RCC. It was the most stable miRNA according to the comprehensive analysis of RefFinder among the 120 studied, and also according to the Delta Ct method and NormFinder. Moreover, it had a good expression level in urine (mean Ct value in RCC patients and healthy controls = 29.97) and no differences were observed between RCC patients and controls, both in the screening and the validation cohorts. Noticeably, miR-20a-5p has not been related to RCC in previous studies. Following a similar strategy to that presented herein, we identified miR-29c-3p as robust normalizer for urine miRNA studies in bladder cancer [17], what may allow the performance of comparable studies among different laboratories to achieve the direct translation of miRNA studies to daily clinical practice with diagnostic/staging purposes.

Once the best miRNA normalizer was ascertained, we aimed to identify a novel profile of miRNAs in urine able to distinguish RCC patients and controls. We analyzed the expression of 179 miRNAs in a screening cohort of 16 ccRCC patients and 16 healthy controls and we obtained a predictive model for ccRCC that includes 3 miRNAs as predictors: miR-200a-3p, miR-34a-5p and miR-365a-3p. In addition to these three miRNAs, we identified let-7d-5p and miR-205-5p as able to distinguish ccRCC patients from controls. A strong correlation among these miRNAs was observed. Next, we validated this ccRCC profile in an independent cohort of 51 patients (29 ccRCC, 16 papRCC and 6 chrRCC) and 32 controls, achieving an AUC of 0.65 (95% CI [0.51, 0.79], *p* = 0.043). These miRNAs did not seem to be specific to any RCC subtype, as no differences were evidenced among ccRCC, papRCC and chrRCC patients. In addition, their dysregulation may be specific to RCC, as their expression level was similar in a small cohort of benign renal angiomyolipomas and in controls. Finally, the upregulation of miR-200a-3p, let-7d-5p, miR-205-5p, miR-34a-5p and miR-365a-3p in the urine of RCC patients was not influenced by tumor size, suggesting a binary regulation where the presence of tumor might be sufficient to increase miRNA expression. Nonetheless, this evidence should be verified in a larger and independent cohort of RCC patients.

miR-200a has been previously related to RCC and has been proposed as biomarker for early stage RCC in serum, urine and RCC cell lines [19]. In addition, Fedorko et al. [20] also evidenced an increase in let-7d in the urine of ccRCC patients. None of the other dysregulated miRNAs found in our study subject has been previously related to RCC.

Lastly, we aimed to find a profile of dysregulated miRNAs in the urine of RCC patients before and after surgery with potential follow-up value. We identified five miRNAs with increased expression before surgery: let-7d-5p, miR-152-3p, miR-30c-5p, miR-362-3p and miR-30e-3p, which showed a strong correlation. Once validated in a larger and external cohort of RCC patients, these miRNAs may be useful as follow-up markers of disease status after surgery. Song et al. [21] evidenced a decrease in the expression of mir-30c-5p in the urine exosomes of ccRCC patients compared to controls and other urologic cancer patients (bladder and prostate cancer). miR-152-3p, miR-362-3p and miR-30e-3p have not been previously related to ccRCC.

To delve into the biological mechanism(s) regulated by these miRNAs, we identified their targets and their relation to the *renal cell carcinoma* pathway. We found that most miRNAs are potential regulators of several components along the *renal cell carcinoma* pathway. Remarkably, all miRNAs but miR-205-5p and let-7d-5p have targets involved in the *renal cell carcinoma* pathway that have been validated in previous studies. This fact reinforces the potential regulatory role of these miRNAs in RCC patients. In addition, all nine dysregulated miRNAs identified have predicted targets. Additional in vitro studies in cell cultures and in vivo in animal models will prove the final regulation of each predicted target and will shed light on the degree of participation of each miRNA in the global regulatory mechanisms in RCC patients. Henceforth, we will discuss several of these regulations in detail.

One of well-known signaling pathways related to cancer is the PI3K/AKT/mTOR pathway which has been proposed for the development of specific PI3K, Akt, and mTOR inhibitors in cancer treatment [22]. This pathway regulates various hallmarks of cancer, such as proliferation, survival, angiogenesis, invasion, metastasis, autophagy, and epithelial-to-mesenchymal transition. The AKT protein is central to the proliferation and survival of normal and cancer cells. Hence, it has been proposed as therapeutic target for different types of cancer [23,24]. miR-365a-3p has AKT1 as a validated target and miR-362-3p as a predicted target. Another important protein of the PI3K/AKT/mTOR pathway, PI3K, is deregulated in a wide spectrum of human cancers and the inhibition of PI3K can result in both decreased cellular proliferation and increased cellular death [25]. Interestingly, several of the dysregulated miRNAs identified have PI3K or the PI3K regulatory subunit as validated (miR-30c-5p, miR-362-3p) or predicted target (miR-34a-5p, miR-365a-3p, miR-205-5p).

The MAPK pathway is a complex interconnected signaling cascade frequently involved in oncogenesis, tumor progression, and drug resistance [26]. Herein, MAPK is a validated target (miR-34a-5p, miR-30c-5p) or predicted target (miR-205-5p, miR-30e-3p) of several miRNAs related to RCC.

In sum, the targets of the dysregulated miRNAs identified in RCC patients seem to participate in relevant pathways of cancer development, although scarce studies have been conducted in RCC. However, the overall regulatory outcome of these miRNAs is hard to ascertain. The regulation of human biological pathways is overly complex since one miRNA usually targets many mRNAs in the same pathway and every mRNA is targeted by many miRNAs to ensure a fine-tuned global regulation. Furthermore, the targets regulated by each miRNA may have opposite functions, which presents controversy as to whether the final outcome of a miRNA would then be oncogenic or tumor suppressive. It is now known that depending on the balance between miRNA-mediated upregulation or downregulation of oncogenic and tumor suppressive pathways, as well as the effects of the miRNA on cancer-immune system interactions and various other tumor-modifying extrinsic factors, the miRNA may produce an overall net oncogenic or net tumor suppressive effect [27]. Consequently, in our particular scenario the degree of relevance of each single miRNA in the regulation of every targeted mRNA in the *renal cell carcinoma* pathway should be empirically validated in animal models through a complex experimental set-up, what should be achieved in future studies.

The evaluation of additional stably expressed miRNAs proposed by RefFinder may have rendered other potential normalizers for urine miRNA studies in the context of RCC, what represents a limitation of our study. Nonetheless, our results confirm previous findings and reinforce the use of miR-20a-5p as a normalizer. Regarding the dysregulated miRNA profiles identified in RCC patients, another limitation is the rather small sample size studied, consistent with it being a discovery-stage study. The validation of our results in an independent external cohort of RCC patients prospectively recruited and followed would definitively reinforce our findings. The strengths of our study are a thorough evaluation of patients at inclusion and during follow-up, the inclusion of healthy volunteers with a clinical evidence of absence of renal or bladder disorders and the validation in an independent cohort of patients and controls.

## 4. Materials and Methods

### 4.1. Patient Recruitment

For the initial screening stage, 16 ccRCC patients who underwent curative or cytoreductive surgery for sporadic, nonmetastatic (M0) or metastatic, unilateral tumor were recruited between 2015 and 2019 and prospectively followed for one year at La Fe University and Polytechnic Hospital (Valencia, Spain). Sixteen age- and sex-matched healthy volunteers (control group) who underwent an ultrasound scan to rule out the presence of kidney or bladder malignancies or other alterations were also recruited. For the validation stage, an independent cohort of 51 RCC patients were recruited before surgery (29 ccRCC, 16 papRCC and 6 chrRCC) together with 13 benign renal angiomyolipomas and 32 healthy volunteers as controls.

Pre-operative clinical staging was performed through physical examination and CT scans of the chest, abdomen and pelvis. The tumor histological classification was performed according to the Fuhrman grade. Demographic and clinical data were collected.

The exclusion criteria were lack of informed consent, absence of histological confirmation and presence of other malignancies.

Informed consent was obtained from all participants according to protocols approved by the ethics review board at La Fe University and Polytechnic Hospital. The study was performed according to the declaration of Helsinki, as amended in Edinburgh in 2000.

### 4.2. Urine Collection

A first morning urine sample of 25 to 50 mL was collected in sterile containers from all participants at recruitment (t_0_) and kept at 4 °C until processing. In all RCC patients, a second urine sample was additionally collected fourteen weeks after surgery (t_1_). Urine was centrifuged at 805× *g* for 5 min at 4 °C to remove cellular debris and supernatant was aliquoted and frozen at −80 °C until analyzed. The concentration of creatinine in urine was measured by clinical laboratory standardized methods.

### 4.3. RNA Isolation and cDNA Synthesis from Urine

Total urine RNA (including miRNAs) was isolated from 600 µL of urine using the miRNeasy Mini Kit (Qiagen, Hilden, Germany) following manufacturer’s instructions with several modifications optimized by our group [28]. Briefly, 200 µL of cell-free urine were transferred to a tube with 1 mL Qiazol (Qiagen) and 1 µL carrier (1 µg/µL yeast RNA, Invitrogen, ThermoFisher Scientific, Waltham, MA, USA). This step was performed in three independent tubes for each sample. In one of the three tubes 1 µL spike-in mix (UniSp2/4/5, Vedbaek, Exiqon, Denmark) was also added and each tube was gently mixed. After a 5 min incubation at room temperature, 200 µL chloroform were added to each tube, and centrifuged at 12,000× *g*, 15 min at 4 °C to allow phase separation. Ethanol in a proportion of 1.5:1 (volume:volume) was added to the liquid phase. The three tubes containing the urine sample from the same individual were pooled in one single column in order to increase the final RNA yield. Then, 4 cleaning steps with the buffers supplied in the kit were performed. Total RNA was finally eluted in 50 µL of DNase/RNase-free sterile distilled water.

The concentration and purity of the RNA was assessed by spectrophotometric quantification with the NanoDrop ND-1000 (Thermo Fisher Scientific). RNA was stored at −80 °C until used.

In the initial screening stage where predesigned panels were used, cDNA was obtained from 5 µL of urine RNA with the miRCURY LNA RT Kit (Qiagen) according to the supplied protocol in a final reaction volume of 25 µL. Due to the addition of a RNA carrier during the isolation, the final RNA yield includes the RNA isolated from urine plus the carrier RNA. Therefore, urine RNA retrotranscription was based on volume (µL) rather than RNA quantity (ng), according to the suppliers’ recommendations. In the validation stage, where the expression level of selected miRNAs was conducted, cDNA was obtained from 2 µL of urine RNA using the same technology (final reaction volume 10 µL). In all cases, the reaction mix containing RNA, enzyme, buffer, RNAse-free water and UniSp6 RNA spike-in template, was incubated 60 min at 42 °C followed by 5 min at 95 °C for reverse transcriptase inactivation. Reactions were carried out in a thermocycler TC-412 (Techne, Staffordshire, UK).

### 4.4. miRNAs Quantification

In the screening stage, 32 urine cDNA samples (16 samples from t_0_ and 16 paired samples from t_1_) from RCC patients and 16 from healthy controls were analyzed. In them, a total of 179 miRNAs were quantified using the commercially predesigned Serum/plasma Focus microRNA PCR Panel V4 (Exiqon). This panel contains 179 miRNAs commonly found in human plasma and serum according to the manufacturer´s in-house analyses of miRNA expression in blood, serum and plasma samples, as well as on the limited number of peer-reviewed published papers available. The list of all the quantified miRNAs is detailed in Table S1. miRCURY LNA SYBR Green Master Mix (Exiqon) was used as a fluorophore, according to manufacturer´s indications. Briefly, cDNA (dilution 1/40), water and PCR master Mix (which includes SYBR Green) were added to a 384-well PCR plate supplied that includes the LNA^TM^ primer sets in a final reaction volume of 10 µL. Furthermore, each panel included the following internal controls: 5 synthetic RNAs of the RNA spike-in-kit aimed to monitor the RNA isolation and cDNA synthesis; an inter-plate calibrator in triplicate; and a negative control to evaluate qPCR performance. qPCR reactions were performed as follows: a polymerase activation/denaturation cycle of 10 min at 95 °C followed by 55 cycles of 10 s at 95 °C and 1 min at 60 °C with a ramp-rate of 2.2 °C/s. All RT-qPCR reactions were conducted in a LightCycler 480 II (Roche, Penzberg, Germany). In the validation stage, selected miRNAs were quantified using specific LNA PCR primer sets (Qiagen) in a total of 102 urine samples from RCC patients (51 patients at t_0_ and t_1_), 13 from renal benign angiomyolipomas and 32 from healthy controls.

### 4.5. Selection of Candidate miRNA Normalizers and Analysis of Their Stability

To normalize the expression level of each miRNA, the best candidate with the highest stability and the lowest biological variance over the entire range of samples being investigated (RCC and controls) was selected. With that aim, all miRNAs with a mean Ct < 35 in RCC and controls were scrutinized. To select the best normalizer, the comprehensive tool RefFinder was employed, which integrates the computational programs Genorm, BestKeeper, the comparative Delta Ct method and Normfinder (available online: https://www.heartcure.com.au/for-researchers/ (accessed on 13 April 2018)) (2012_miRDeepFinder: a miRNA analysis tool for deep sequencing of plant small RNAs). Normalization of miRNA expression was conducted with the ^∆∆^Ct method.

### 4.6. Identification of miRNAs’ Targets

Once the dysregulated miRNAs were selected, their validated and predicted target proteins related to RCC were identified using the databases miRWalk 2.0 (available online: http://zmf.umm.uni-heidelberg.de/apps/zmf/mirwalk2/ (accessed on 5 February 2019)) and Kyoto Encyclopedia of Genes and Genomes (KEGG) (available online: https://www.genome.jp/kegg/ (accessed on 5 February 2019)). miRWalk 2.0 combines information from 12 existing miRNA-target prediction programs (DIANA-microTv4.0, DIANA-microT-CDS, miRanda-rel2010, mirBridge, miRDB4.0, miRmap, miRNAMap, doRiNA, i.e., PicTar2, PITA, RNA22v2, RNAhybrid2.1 and Targetscan6.2). Subsequently, the targets obtained with miRWalk 2.0 were integrated within the *renal cell carcinoma* pathway from KEGG. These miRNAs could bind to target mRNAs on the 3´UTR and 5′UTR regions and, consequently, these may regulate the expression of proteins involved in the *renal cell carcinoma* pathway.

### 4.7. Statistical Analysis

All statistical analyses were performed using R (version v3.5.1). Numerical variables were summarized as median and interquartile range, and categorical variables as count and percentage. In the screening stage, an elastic net logistic regression model for RCC risk was adjusted using the different miRNA expression levels at inclusion as potential predictors. Selection of the lambda value (controlling variable selection) was performed by 500 repetitions of 10-fold cross-validation. The predictive ability of the model was assessed by estimating an optimism-corrected area under the curve (AUC) for the receiver operator characteristic (ROC) analysis, and using 1000 bootstrap replicates in the screening stage. Next, this AUC was validated in the validation stage. The formula to calculate the risk of RCC in each patient was built with the coefficients rendered by the model for each predictive miRNA. In addition, dysregulated miRNAs between two clinical groups were identified using the Wilcoxon–Mann–Whitney test. Furthermore, a paired Wilcoxon test was applied to identify dysregulated miRNAs in the samples obtained before and after surgery (t_0_ and t_1_). The degree of correlation between the dysregulated miRNAs was assessed with the Spearman test. Results were considered statistically significant at *p* < 0.05.

## 5. Conclusions

In summary, our study is the first report characterizing a reliable normalizer for the analysis of urine miRNAs in RCC patients. miR-20a-5p, being one of the most stably expressed miRNAs in urine of RCC patients and healthy individuals, arises as an optimal reference miRNA that may allow the comparison of future urine miRNA studies as non-invasive biomarkers for RCC diagnosis and monitoring. Using this miRNA as normalizer, we have identified a urine profile of three miRNAs (miR-200a-3p, miR-34a-3p and miR-365a-3p) with potential diagnostic value for RCC and a profile of five miRNAs (let-7d-5p, miR-152-3p, miR-30c-5p, miR-362-3p and miR-30e-3p) dysregulated in patients before surgery with respect to fourteen weeks after, with potential follow-up value. All miRNAs are deeply involved in the *renal cell carcinoma* pathway. Once validated in a larger and external cohort, these non-invasive biomarkers may improve RCC diagnosis and follow-up, reducing the performance of harmful and expensive procedures.

## Figures and Tables

**Table 1:** Clinical characteristics of the RCC patients and controls studied.

	Screening Stage Cohort		Validation Stage Cohort		
	Patients N = 16	Controls N = 16	Patients N = 51	Controls N = 32	Angiomyolipomas N = 13
Age, y	67.5 (61.25–71)	68 (59.50–71.25)	63 (52–69)	62.5 (51–71.5)	61 (45–70)
Male sex, N (%)	13 (81.25%)	13 (81.25%)	29 (56.86%)	21 (65.63%)	1 (7.69%)
Urine creatinine, mg/dL	80.7 (61.7–147.9)	85.7 (57.1–111.9)	70.6 (42.7–122.7)	96.75 (62.6–154.1)	64 (53.15–109.3)
RCC Tumor type, N (%)					
ccRCC	16 (100%)	-	29 (56.86%)	-	-
papRCC	-	-	16 (31.37%)	-	-
chrRCC	-	-	6 (11.77%)	-	-
Tumor Stage, N (%)					
I	13 (81.25%)	-	41 (80.39%)	-	-
II	2 (12.5%)	-	2 (3.92%)	-	-
III	1 (6.25%)	-	6 (11.77%)	-	-
VI	-	-	2 (3.92%)	-	-

Continuous variables are presented as median and interquartile range and categorical variables are presented as count and percentage. ccRCC, clear-cell renal cell carcinoma; papRCC, papillary renal cell carcinoma; chrRCC, chromophobe renal cell carcinoma.

**Table 2:** Correlation among the miRNAs dysregulated in ccRCC patients before surgery (t_0_) compared to controls. Spearman correlation coefficients between two miRNAs are detailed. Significant *p*-values are depicted in bold.

	miR-200a-3p	let-7d-5p	miR-205-5p	miR-34a-5p	miR-365a-3p
miR-200a-3p	1	0.458 (**0.008**)	−0.229 (0.207)	0.375 (**0.034**)	0.474 (**0.006**)
let-7d-5p		1	0.198 (0.276)	0.645 (**0.0001**)	0.411 (**0.019**)
miR-205-5p			1	0.570 (**0.001**)	0.234 (0.197)
miR-34a-5p				1	0.435 (**0.013**)
miR-365a-3p					1

**Table 3:** Correlation among dysregulated miRNAs in ccRCC patients before (t_0_) and after surgery (t_1_). Spearman correlation coefficients between two miRNAs are detailed. Significant *p*-values are depicted in bold.

	let-7d-5p	miR-152-3p	miR-30c-5p	miR-362-3p	miR-30e-3p
let-7d-5p	1	0.467 **(0.007**)	0.786 (**0.0001**)	0.432 (**0.013**)	0.600 (**0.0001**)
miR-152-3p		1	0.622 (**0.0001**)	0.211 (0.247)	0.499 (**0.004**)
miR-30c-5p			1	0.446 (**0.011**)	0.671 (**0.0001**)
miR-362-3p				1	0.519 (**0.002**)
miR-30e-3p					1

**Table 4:** Validated and predicted targets of the miRNAs dysregulated in RCC patients. These target proteins were identified using miRWalk 2.0 and were further integrated within the renal cell carcinoma pathway from KEGG. Validated targets are those that have been empirically validated to be regulated by a miRNA. Predicted targets are those that have been theoretically estimated based the free binding energy between a miRNA and a putative target mRNA sequence.

		*Renal cell carcinoma* Pathway	
miRNA	Sequence	Validated Targets	Predicted Targets
**Comparison: RCC Patients before Surgery and Controls**			
miR-200a-3p *	uaacacugucugguaacgaugu	GRB2	HGF, TGFB2, CUL2
miR-34a-5p *	uggcagugucuuagcugguugu	FH, GRB2, MAP2K1, MAPK3, MET, VEGFA	PIK3CB, PTPN11, ARNT
miR-365a-3p *	uaaugccccuaaaaauccuuau	AKT1, KRAS, RAC1	TGFB3, PIK3R3
miR-205-5p	uccuucauuccaccggagucug	-	VEGFA, MAPK3, PIK3CG, TCEB1, EGLN2, VEGFA
let-7d-5p	agagguaguagguugcauaguu	-	BRAF, HGF
**Comparison: RCC Patients before (t_0_) and After Surgery (t_1_)**			
let-7d-5p	agagguaguagguugcauaguu	-	BRAF, HGF
miR-152-3p	ucagugcaugacagaacuugg	KRAS, SOS2, TGFA	ETS1, SOS1, TGFB2, EPAS1, SLC2A1, MET, JUN, PAK3, GRB2, GAB1
miR-30c-5p	uguaaacauccuacacucucagc	CUL2, EP300, ETS1, GAB1, MAP2K1, PIK3R2, RAC1, RAP1B, TGFA	PIK3CB, PIK3CD, EGLN1, TCEB1
miR-362-3p	aacacaccuauucaaggauuca	ARNT, CRK, ETS1, PAK6, PIK3CG, PIK3R1, RAPGEF1, VEGFA	AKT2, MET, CUL2, RAP1B, CDC42, RBX1
miR-30e-3p	cuuucagucggauguuuacagc	CRK, KRAS, SOS2	GRB2, EGLN1, RBX1, MAP2K2

* miRNAs included in the multivariable elastic net logistic regression model of ccRCC before surgery (t_0_) compared to healthy controls. miRNA sequences detailed in accordance with miRBase 22.1.

## Data Availability

The data that support the findings of this study are available from the corresponding author, [P.M.], upon reasonable request.

## Supplementary Materials

The following are available online at https://www.mdpi.com/article/10.3390/ijms22157913/s1, Table S1: miRNAs and internal controls included in the Serum/plasma miRNA PCR Panel V4 (Exiqon).
